# Analysis of craniocerebral injury in facial collision accidents

**DOI:** 10.1371/journal.pone.0240359

**Published:** 2020-10-26

**Authors:** Jie Tian, Chuntao Zhang, Qun Wang

**Affiliations:** College of Automobile and Traffic Engineering, Nanjing Forestry University, Nanjing, Jaingsu, China; Tongii University, CHINA

## Abstract

Considering that the Pc-Crash multibody dynamics software can reproduce the accident process accurately and obtain the collision parameters of pedestrian heads at the moment of head landing, the finite element analysis method can accurately analyze the injury of the pedestrian head when the boundary conditions are known. This paper combines the accident reconstruction method with the finite element analysis method to study the injury mechanism of pedestrian head impact on the ground in vehicle pedestrian collision accidents to provide a theoretical basis for pedestrian protection and the improvement of vehicle shapes. First, a real-life vehicle pedestrian collision is reproduced by Pc-Crash. The simulation results show that the rigid multibody model can accurately simulate the scene of the accident, then the speed and angle of the pedestrian head landing moment can be obtained at the same time. Second, the finite element model of human heads with a detailed facial structure is established and verified. Finally, the collision parameters obtained from the accident reconstruction are used as the boundary conditions to analyze the collision between the pedestrian head and the ground, and the biomechanical parameters, such as intracranial pressure, von Mises stress, shear stress and strain, can be determined. The results show that the stress wave will propagate inside and outside the skull and cause stress concentration in the skull and the brain tissue to varying degrees after the pedestrian head strikes the ground. When the stress exceeds a certain limit, it will cause different degrees of brain tissue injury.

## 1 Introduction

With the development of automobile technology, transportation has become more convenient and efficient, but it also leads to more traffic accidents, whose contributing factors may be the driver’s demographic or behavioral characteristics, vehicle’s technical characteristics, roadway conditions and environmental factors. Therefore, injury crashes become a major concern of researchers, policy-makers, and the public. In order to mitigate the enormous economic and emotional burden brought to the society by traffic accidents, people are devoted to reducing the frequency and severity levels of traffic crashes. Most of the traditional collision frequency studies were carried out on historical crash data using discrete outcome models [1–5]. In addition, the pedestrian and bicycle injuries from statistical point of view were also studied [6–8].

However, in the traffic system, pedestrians are in the most vulnerable position. Compared with other road users, pedestrians usually face a greater risk of injury and death. The death rate of pedestrians due to traffic accidents is the highest, accounting for 26% of the total number of deaths [9]. In 2019, the number of deaths due to traffic accidents in China was approximately 100,000, of which the total number of pedestrian deaths was approximately 25000, accounting for 25% of the total number of deaths in traffic accidents in the whole year [10]. According to a statistical report of the World Health Organization (WHO), the head is the most vulnerable part of the body in vehicle pedestrian accidents, accounting for 26.5%-39.3% of traffic injuries considering all parts of the human body [11].

In an accident, the main form of damage is impact injury followed by the fall injury in the secondary collision stage. The secondary injuries include falling, rolling on the ground and striking other objects. Generally, serious injury to pedestrians is not caused by the second collision but when the pedestrians’ head directly impacts the ground, which may produce serious brain injury. At present, domestic and international research primarily focus on the pedestrian-vehicle collision, and the research on pedestrian-ground collision is limited. Therefore, in view of this oversight, it is necessary to analyze the damage mechanism and corresponding damage risk of pedestrian head impact on the ground through more in-depth research.

In general, scholars often use a rigid multibody model to study the dynamic response of pedestrians during and after the collision [12–14]. In 2011, Simms et al. [12] performed a collision simulation of a medium-sized male pedestrian model and a female pedestrian model with six types of vehicles at a speed of 25–35 km/h. These researchers found that vehicles with high engine hood edges, such as sport utility vehicle (SUV), were more likely to cause the direct collision between the human head and the ground than those with lower engine hood edges. In 2012, Elliott et al. [13] found that 94% of 72 simulation cases had pedestrian head-to-ground collision. In 2013, Gupta et al. [14] simulated the impact of a medium-sized man, a woman and a 6-year-old child with a car and an SUV model, respectively. It was found that lowering the front contour of the car and raising the front contour of the SUV can prevent the pedestrian head from directly hitting the ground, but the mechanism of this situation has not been elucidated. However, due to the simplicity of the multibody model, it is difficult to obtain the specific biomechanical parameters of head injury; therefore, there are limitations to using the multibody model to study pedestrian head landing injuries.

At present, finite element method has been widely used in automotive engineering industry [15, 16]. And domestic and international researchers began to use the finite element method to study the mechanism of head landing injury. In 2013, Li et al. [17] established a finite element model of the child head and then compared it to the experimental data of a child cadaver, and they used the finite element model to further study the influence of different drop heights and surface stiffness on the dynamic response of the head. In 2014, Tamura a et al. [18] used the finite element method to reconstruct the ground collision. The results showed that the kinematics and dynamics of pedestrians after the collision were not easy to predict. The study also suggested that the risk of sustained traumatic brain injury (TBI) due to pedestrian contact with the ground should be the focus of future research. In 2015, Wang Cong [19] dropped the finite element model of the head from different heights to a ground surface composed of different materials to study the influence of different drop heights and ground stiffnesses on brain injury. In addition, under the same conditions, the head model was dropped to the ground in the front and side, respectively, to analyze the influence of the impact site on brain injury.

In summary, scholars have primarily studied vehicle pedestrian collisions and pedestrian head landing injuries. For the former, the studies primarily adopted the rigid multibody model method. However, the limitation of the rigid multibody is that it is difficult to obtain the specific biomechanical parameters of the head, and it is difficult to analyze the specific mechanism of head injury. For the latter, the finite element analysis method was used to study the damage caused to the head by falling from different heights, but they did not combine their research with studies of real-life vehicle pedestrian collision accidents. Therefore, this paper combines the above two methods to study the pedestrian head landing injury mechanism in a real-life vehicle pedestrian collision accident.

The main contribution of this paper is to provide a set of feasible research methods for the pedestrian head landing injury in vehicle pedestrian collision accidents. Specifically, the collision parameters are obtained through accident reconstruction, and then the pedestrian head landing injury analysis is carried out on this basis. This method can effectively reproduce the scene of the accident, judge the severity of head injury, and analyze the injury mechanism, which can provide a reference for further study of pedestrian head protection and improvement of vehicle head shape.

The article structure is as follows: A real-life vehicle pedestrian collision accident is reconstructed in Section 2. In Section 3, a head finite element model is established and verified, and then the velocity and angle of pedestrian head impact on the ground, obtained from a rigid multibody simulation, are taken as the initial conditions of finite element simulation. Section 4 discusses the results of finite element simulation and analyzes the mechanism of pedestrian head landing injury by observing the propagation path of stress waves. Finally, the conclusions of this study are summarized in Section 5.

## 2 Based on Pc-crash instance reproduction and analysis

In this section, based on a real-life accident example, the collision process is simulated by Pc-Crash, and the kinematics and dynamics of the collision are analyzed.

### 2.1 Accident overview

**Fig 1:**
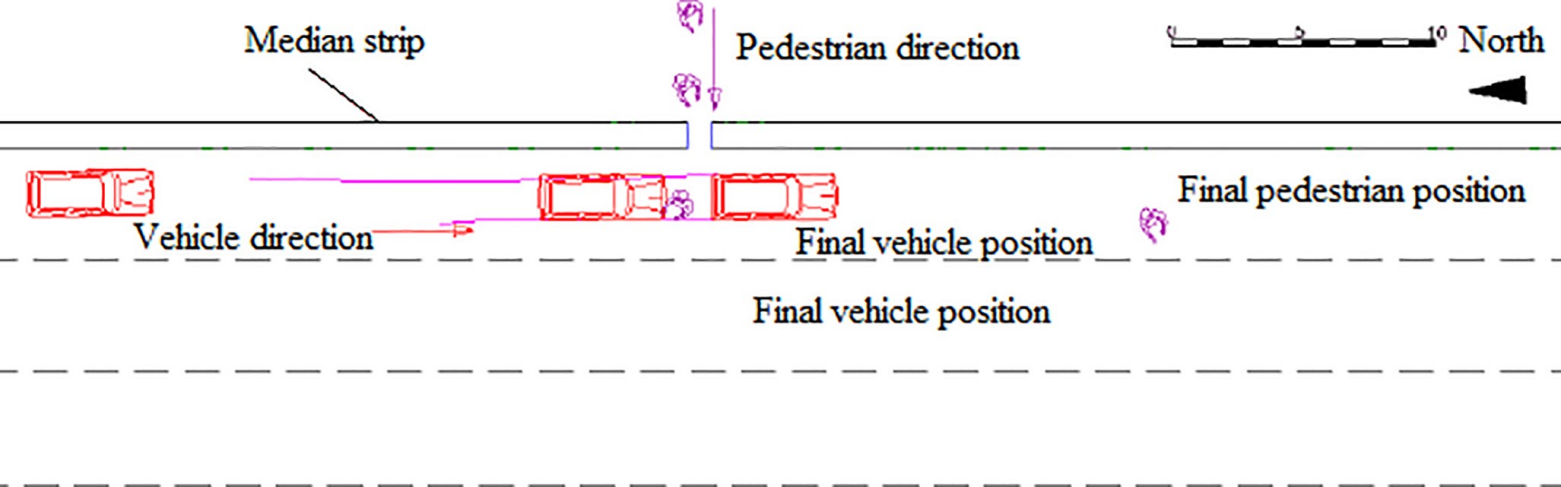
Final location of the accident.

The drawing of the accident scene is presented in Fig 1. One day, a van collided with a pedestrian crossing the green belt in the middle of the road when it reached a certain section. After the collision, the pedestrian eventually fell to the ground and stopped in front of the vehicle, as shown in Fig 1. In the whole collision process, the length of the emergency brake imprint was 10.6 m; the final relative distance between the vehicle and the pedestrian was 11.3 m; and the pedestrian throw distance was 19 m. The pedestrian eventually died with forehead comminuted fractures and severe craniocerebral injury.

**Fig 2:**
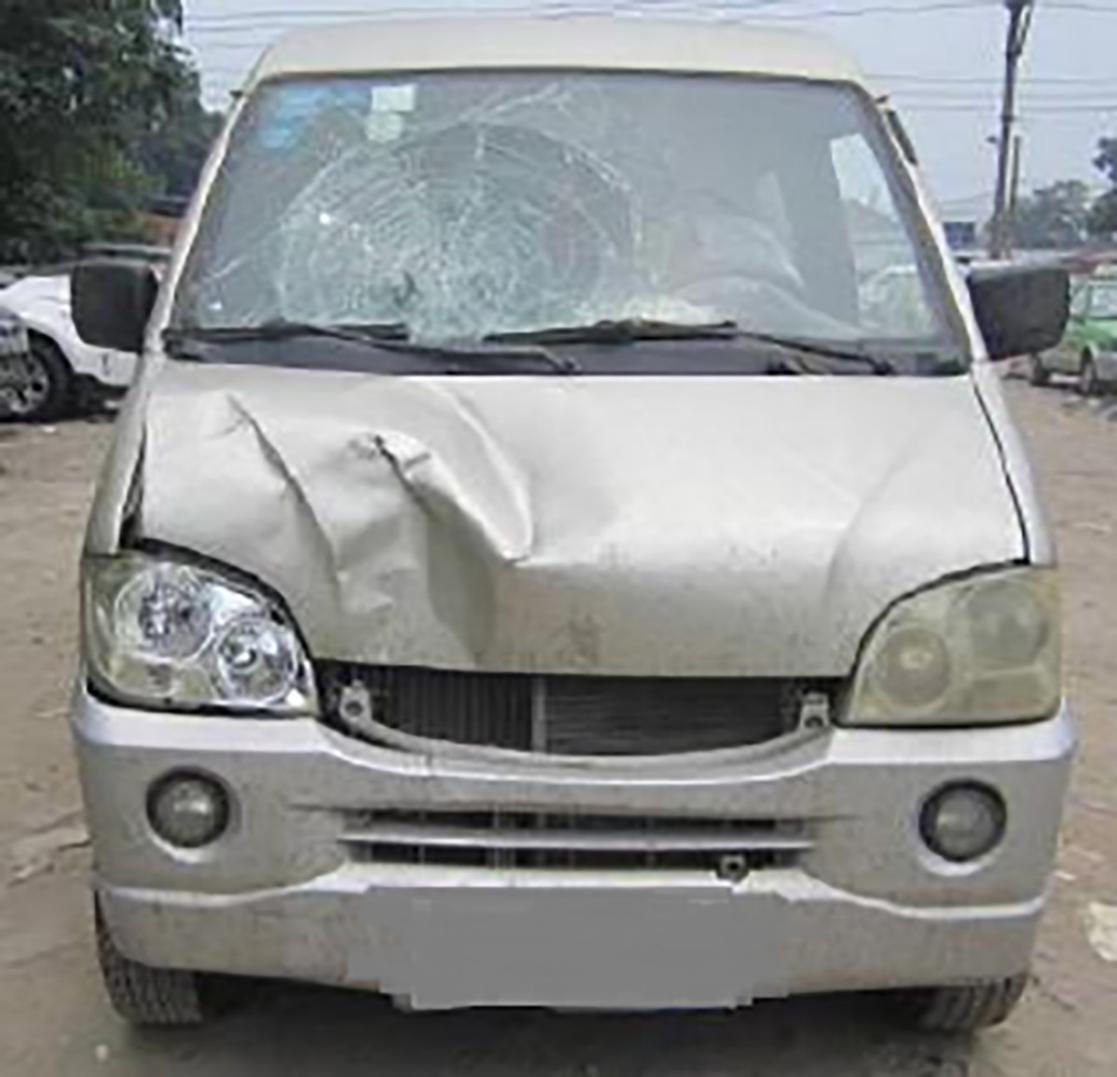
Damage of van.

The damage to the vehicle is shown in Fig 2. The right side of the front bumper was deformed, the right headlamp glass was broken, and the right front end of the engine compartment cover was deformed. The front windshield was broken after the collision with the pedestrian's head, and the collision point exhibited a spiderweb pattern.

### 2.2 Speed calculation

The collision speed is calculated according to the brake imprint. First, according to the energy conservation theorem, the kinetic energy lost by the vehicle in the braking process is equal to the work performed by the friction during braking, and then the following results can be obtained:

$$Fs=\frac{1}{2}mv_{0}^{2}$$ (1)

F=mg(φ±i) (2)

where *F* is the braking friction force, in N; *s* is the braking imprint, in m; *m* is the vehicle mass, in kg; *v*_0_ is the initial speed of the vehicle when the braking imprint occurs, in km/h; *φ* is the road adhesion coefficient, and *φ =* 0.7; *i* is the road slope, and in this instance, *i* = 0; *g* is the acceleration of gravity, in m/s^2^.

Submitting (2) into (1), the instantaneous speed of the vehicle at the beginning of the imprint is

$$v_{0}=\sqrt{2g(\varphi \pm i)s}$$ (3)

In the accident investigation, the brake trace of the vehicle is measured to be 10.6 m. According to the (3), the collision speed of the general-purpose van during the accident is calculated to be 43.4 km/h.

### 2.3 Kinematic analysis

**Fig 3:**
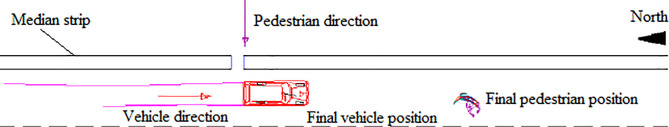
Final position of the vehicle and pedestrian.

The simulation result shows that when the collision speed is 44 km/h, the pedestrian throw distance of is 19.5 m, which is largely consistent with the field data of the accident investigation. The final relative position of the vehicle and the pedestrian is also largely consistent with the field data obtained of the accident investigation, as shown in Fig 3.

**Fig 4:**
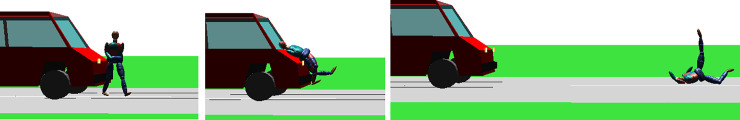
Posture of the pedestrian after collision. **(A)** t = 0.020 s. **(B)** t = 0.100 s. **(C)** t = 1.119 s.

The posture of the pedestrian after the collision is shown in Fig 4. When t = 0.020 s, the right lower leg of the pedestrian is about to collide with the front bumper, which will cause the bumper depression of the van and the pedestrian's fractured right lower leg. When t = 0.100 s, the chest, abdomen and pelvis of the pedestrian collide with the engine hood, resulting in the deformation of the vehicle hood and the fracture of the pedestrian ribs, then the head of the pedestrian hits the front windshield, which causes the breakdown of the vehicle front windshield and the serious brain injury of the pedestrian. At the time of t = 1.119 s, the frontal part of the pedestrian head collides with the ground, causing serious brain injury. The simulation results show that the vehicle pedestrian collision relationship is largely consistent with the damages to the vehicle and the pedestrian in the accident.

### 2.4 Dynamic analysis

**Fig 5:**
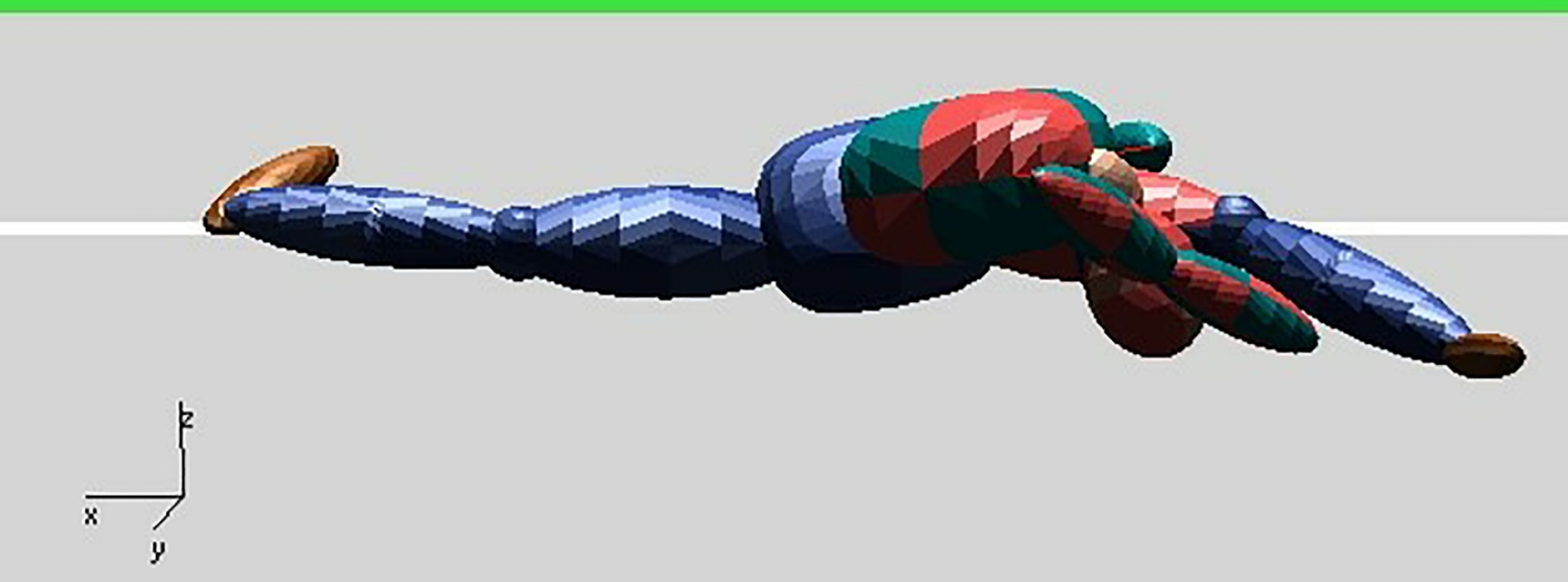
Frontal landing moment.

**Fig 6:**
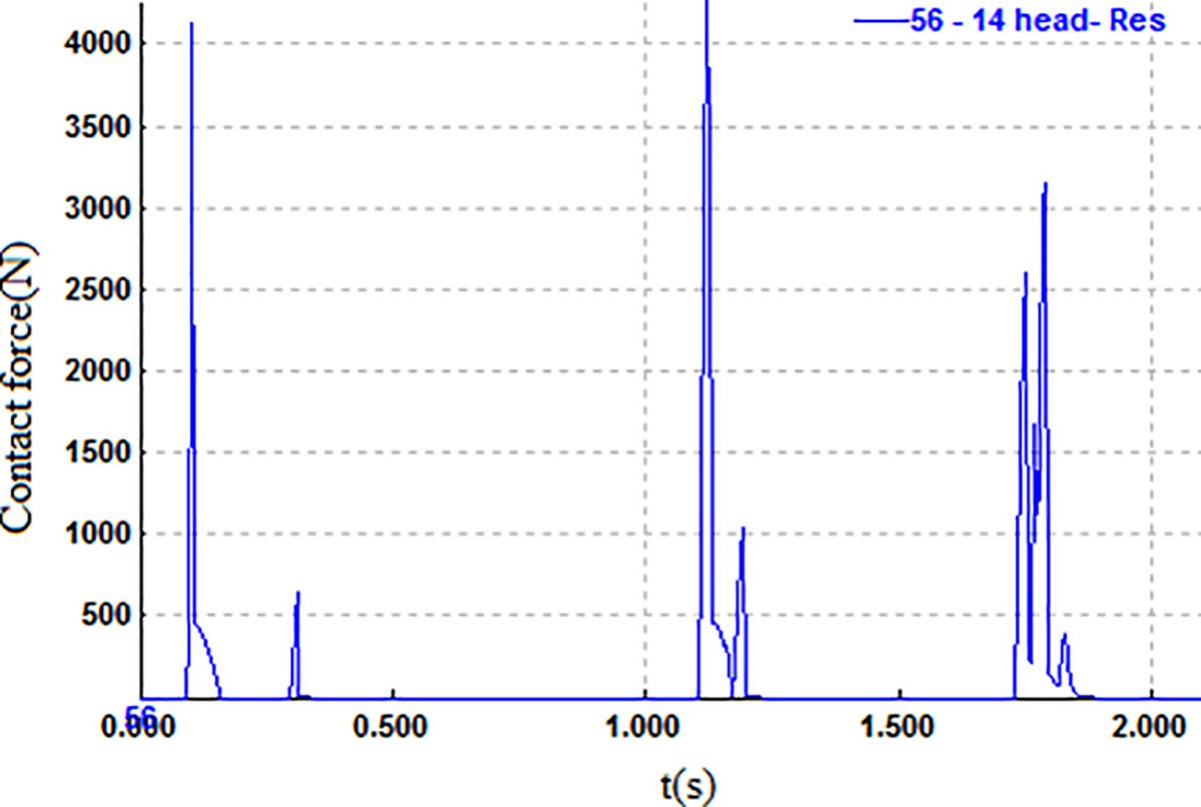
Contact force curve of pedestrian head.

**Fig 7:**
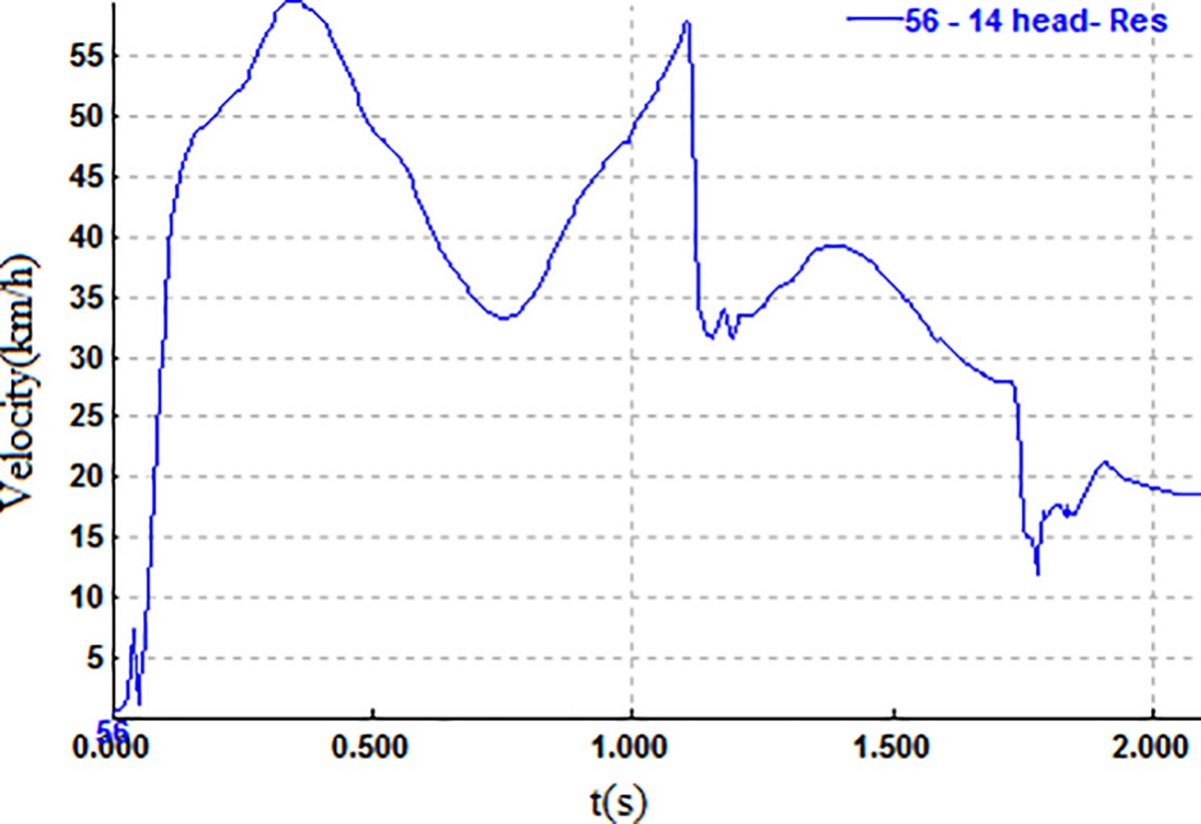
Speed curve of the pedestrian head.

Finally, the pedestrian lands, with the forehead being the contact point, as shown in Fig 5. The relevant parameters when the pedestrian’s head contacts the ground are shown in Figs 6 and 7.

As seen from Figs 6 and 7, the conclusion can be drawn that the moment of the maximum impact force is t = 1.119 s, the contact force is 4256 N, the instantaneous velocity is 11.11 m/s, and the angle between the impact direction and the ground is 87°.

## 3 Finite element analysis of head landing injury

To evaluate the degree of pedestrian head injury, the main observation indices in the finite element analysis are the intracranial pressure, von Mises stress, shear stress and strain. In addition, by observing the propagation process of the stress wave in the pedestrian's head, this approach can not only determine the head landing injury but also has important clinical significance in the development of intracranial injury mechanism.

### 3.1 Modeling of head finite element

To obtain tomographic images of the human head, computed tomography (CT) and magnetic resonance image (MRI) scanning are performed on the head of a 50th percentile adult male. To preprocess the image, the image is imported into the Mimics, the point cloud data is obtained, and the curved surface is generated by parameterization processing. Before fitting the curved surface, the poor points should be detected and removed. Finally, the three dimensional geometric model, which primarily includes the skull, facial bones, cartilage, teeth, meninges, brain, cerebellum and cerebrospinal fluid, is generated by fitting the curved surface with the B-spline curve. Then, the geometric model is meshed, and the model is imported into Hyper Mesh. The unit body type is set to the linear hexahedron unit. Finally, the physical properties of the bone and brain tissue are defined.

**Fig 8:**
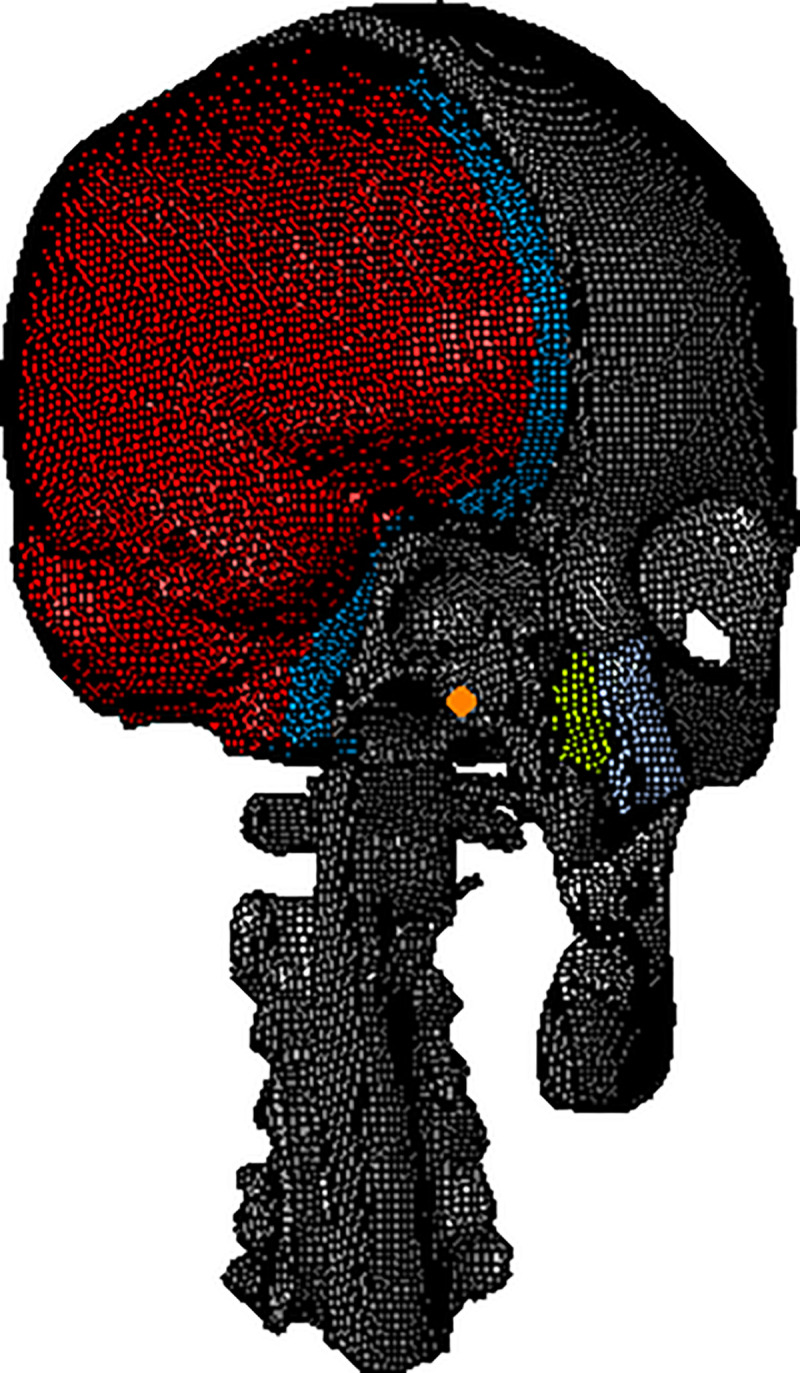
Finite element model of the head.

As shown in Fig 8, in the established head model, the pia mater is attached to the upper surface of the brain tissue, the dura mater is attached to the inner skull surface, and the cerebrospinal fluid layer is located between the pia mater and the dura mater. Finally, the human head finite element model is generated, which contains 403,176 units in total.

### 3.2 Experimental verification of Yoganandan’s head side fall

**Fig 9:**
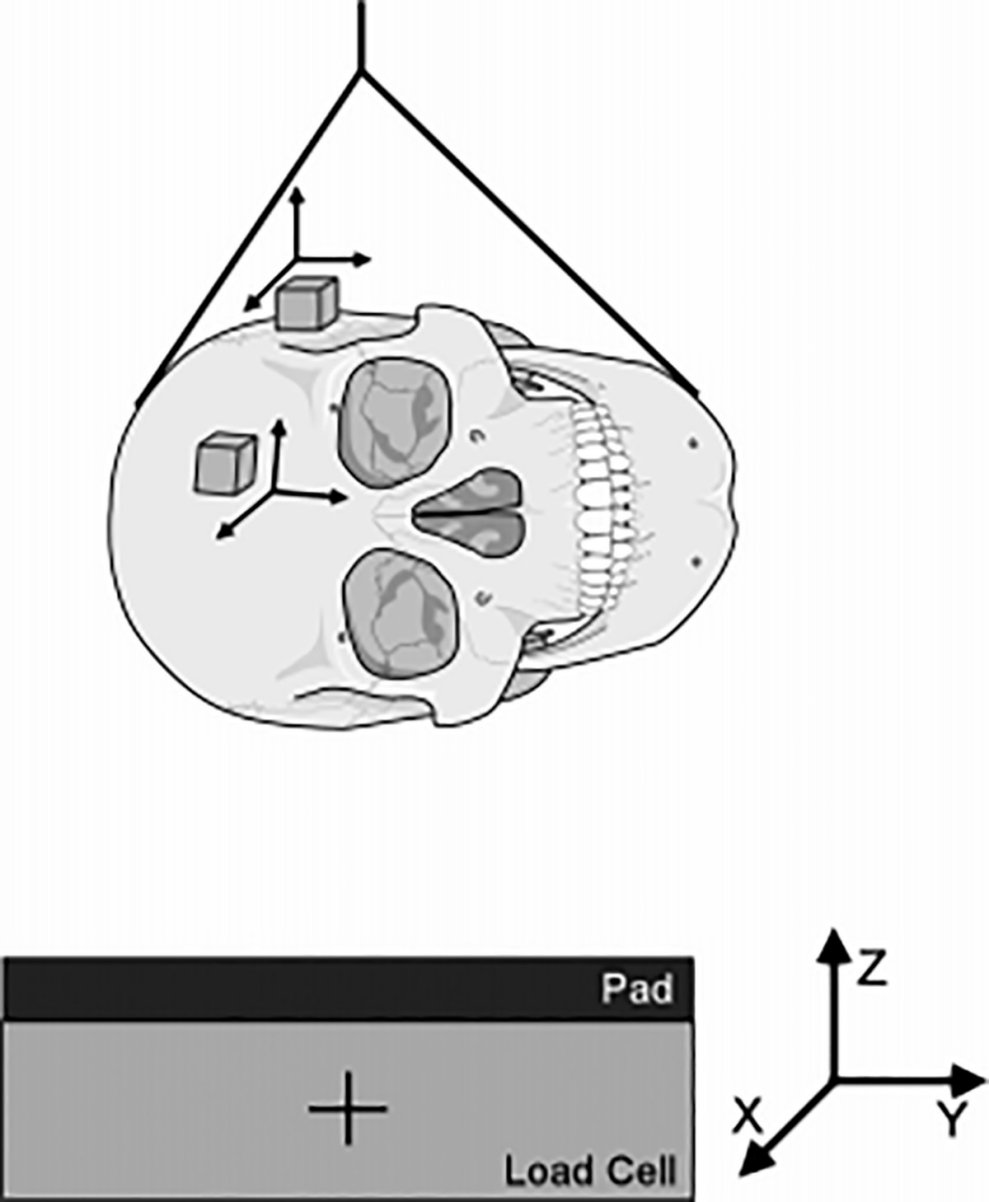
Diagram of Yoganandan cadaver experiment device.

To investigate the biomechanical characteristics of the human head under quasi-static and dynamic loads, Yoganandan [20] performed a side drop test on the heads of 12 corpses without antiseptic treatment in 2004. The six axis load cell placed on the impact platform recorded the time-history curve of the contact force, and the experimental device is shown in Fig 9.

**Fig 10:**
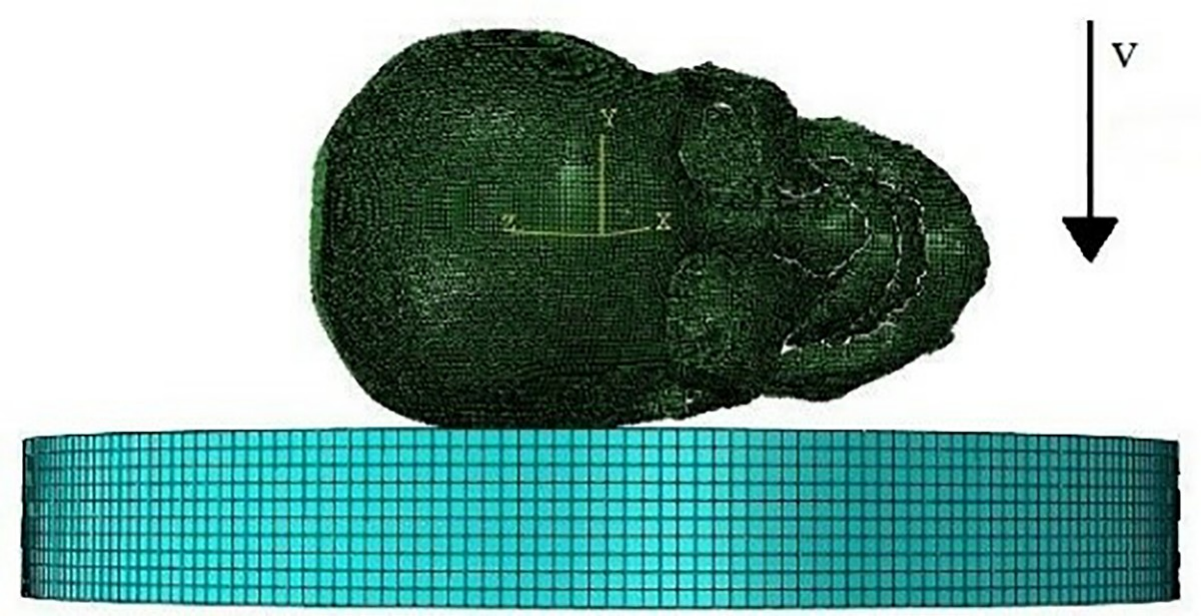
Schematic diagram of impact direction and position in collision simulation.

In this paper, the experimental data of Yoganandan [20] is selected to verify the effectiveness of the head finite element model in side impact. In the simulation process, we fixed the flat plate in the horizontal direction, placed the side of the head model downward parallel to the flat plate, and then dropped the head model from a certain height to the flat plate at a falling speed of 3.5 m/s, 4.9 m/s and 6.0 m/s, respectively, as shown in Fig 10. Finally, the contact force time history curve of the head model falling to the plate was extracted and compared with the results of the cadaver experiment.

**Fig 11:**
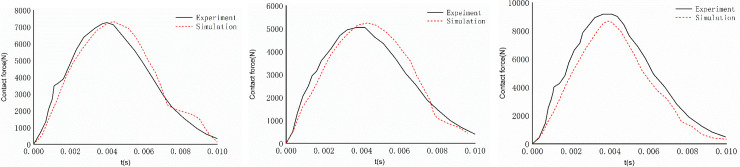
Contact force curve of simulation and cadaver experiment. **(A)** v = 3.5 m/s. **(B)** v = 4.9 m/s. **(C)** v = 6.0 m/s.

Fig 11 shows the contact force time history curves of the simulation result and the cadaver experiment. It can be concluded that regardless of the speed at which the head model falls, the trend of the contact force time-history curve of the cadaver experiment is almost the same as that of the simulation curve. When the falling speed set in the simulation is 3.5 m/s, the maximum contact force of the head model falling to the plate is 5160 N, the maximum value appears in t = 4.3 ms; meanwhile, when the maximum contact force of the head falling to the plate in the cadaver experiment reaches 5069 N, the maximum value appears in t = 4.0 ms. Compared with the experimental results, the time lag of the maximum value in the simulation is 0.3 ms, and the maximum contact force in the simulation is slightly different from the experimental data. When the falling speed set in the simulation is 4.9 m/s, the maximum contact force of the head model falling to the flat plate and the experiment with the corpse are approximately 7218 N, while the maximum value in the simulation appears at 4.2 ms, lagging behind the experimental result by approximately 0.2 ms. When the falling speed set in the simulation is 6.0 m/s, the maximum contact force of the head model falling to the flat plate is 8685 N, the maximum contact force of the cadaver test result is 9215 N, the contact force of the simulation is 5% less than the experimental data, and the landing time is largely the same.

Through the above comparative analysis, it is found that the trend of the simulation results is largely the same as that of the head collision contact force curve obtained from the cadaver experiment, and the difference between the simulation results and the experimental results is small. When analyzing the collision process, the simulation can accurately reflect the intracranial biomechanical response of the pedestrian head, and the model can be used for the evaluation of brain injury during the collision process.

### 3.3 Case of van passenger pedestrian collision

**Fig 12:**
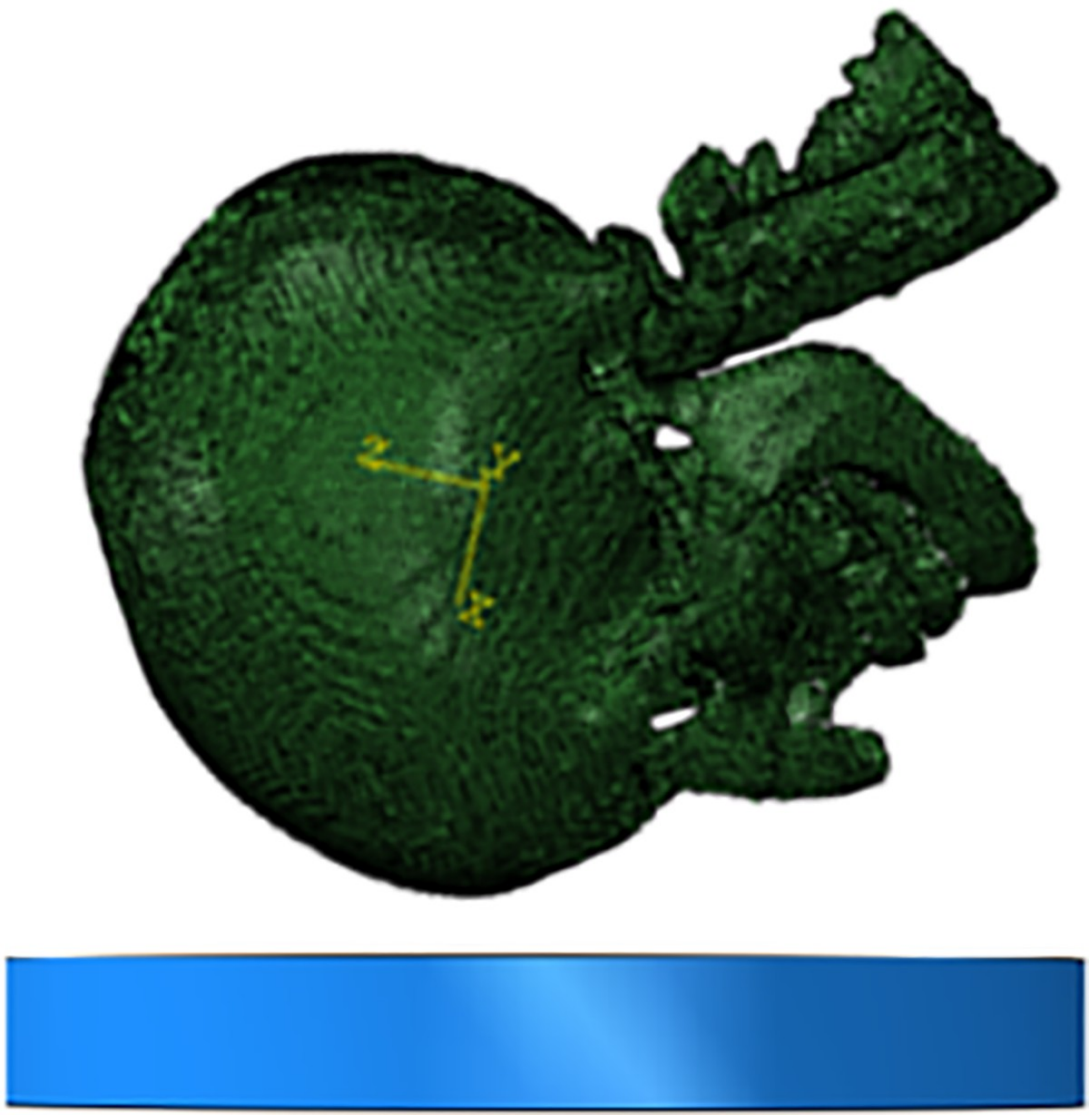
Diagram of frontal landing simulation.

First, we established the road model and set the surrounding and lower surfaces as the fixed boundary conditions according to the actual situation of the road. Then, we set the angle and the speed of the collision between the forehead of the head model and the ground and completed the output setting of other results. The simulation of the head landing on the forehead is shown in Fig 12.

**Fig 13:**
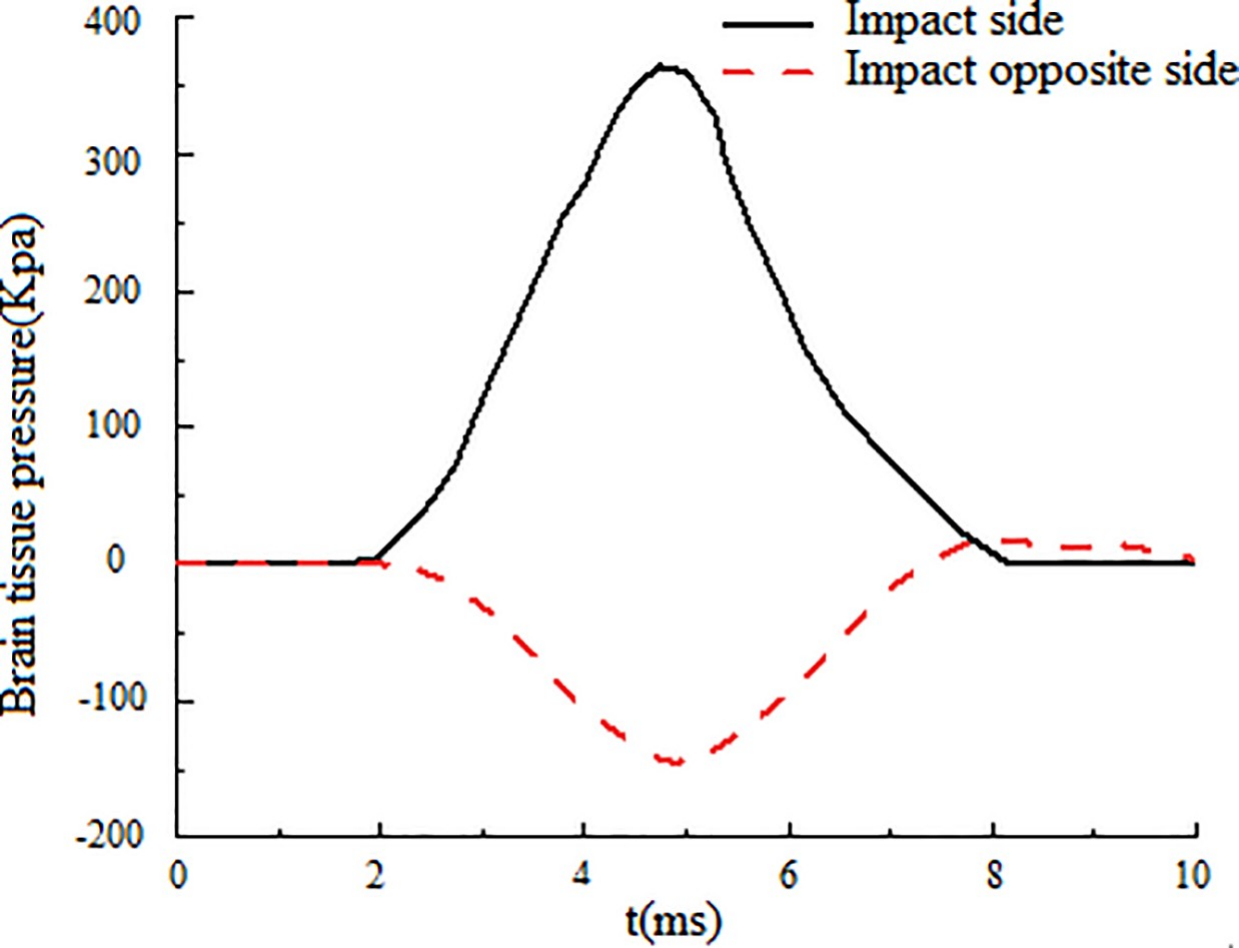
Intracranial pressure of frontal collision varies with time.

Fig 13 shows the curve of intracranial pressure with time when the pedestrian's forehead lands on the ground. During the whole collision process, the intracranial pressure in the frontal part is largely positive, indicating that the brain tissue in the frontal part is in a state of compression, which is caused by inertia.

The peak value of the positive pressure on the collision side is 363 kPa. On the other side of the collision, because the brain tissue and skull are separated to produce tensile force, the whole collision process is largely negative pressure in the early stage, while the peak negative pressure on the opposite side of the collision is -147 kPa. According to the research of Ward et al. [21], when the intracranial pressure of the adult's head is greater than 235 kPa, it will lead to serious brain injury, which is consistent with the situation that the victim has serious brain injury in the pedestrian injury record.

**Fig 14:**
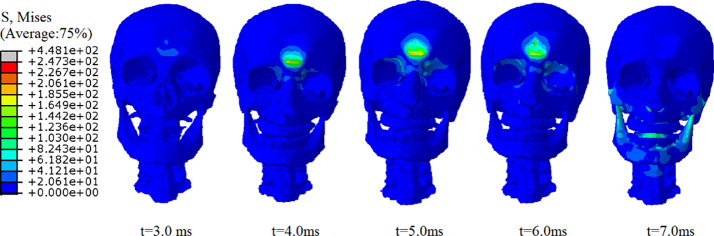
Propagation of stress wave in the skull under frontal collision. **(A)** t = 3.0 ms. **(B)** t = 4.0 ms. **(C)** t = 5.0 ms. **(D)** t = 6.0 ms. **(E)** t = 7.0 ms.

**Fig 15:**
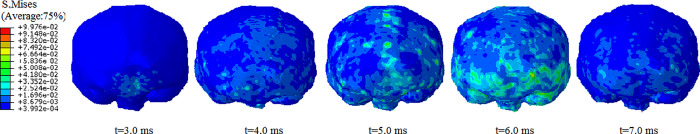
Propagation of stress wave in the brain under temporal collision. **(A)** t = 3.0 ms. **(B)** t = 4.0 ms. **(C)** t = 5.0 ms. **(D)** t = 6.0 ms. **(E)** t = 7.0 ms.

Figs 14 and 15 show the stress wave transmission and distribution in the heads of pedestrians when the pedestrians strike the ground with their foreheads. When pedestrian strike the ground with their foreheads, the movement of the head is suddenly blocked, the skull stops moving, the skull at the frontal collision site is deformed, the impact point on the outside is concave, and the impact point on the inside is convex. Under the action of inertia, the brain tissue keeps the inertia and continues to move along the initial direction of motion. The protuberance of the frontal bone then contacts and collides with the brain tissue. The speed of the brain tissue movement is reduced and the brain tissue at the contact site is deformed.

It can be seen from Fig 14 that when the pedestrian is thrown out, the head of the pedestrian contacts the ground. When t = 3.0 ms, there is no obvious stress concentration at the contact position between the forehead of the pedestrian head and the ground. From t = 4.0 ms to t = 6.0 ms, the stress concentration area at the impact site is increasingly clear. With the continuous impact time, the contact between the forehead and the ground gradually becomes clear and then radiates from the impact site to the surrounding area. Part of the stress continues to move up to the parietal region of the skull and down to the brow, brow arch and supraorbital notch. There is a stress concentration in the supraorbital notch, and then the stress wave also continues to spread to the frontal orbital surface, cribriform plate and tears of the maxillofacial bone. Because the structure of these areas is weak and the end face is narrow, the stress concentration appears in these areas.

In the impacting process, there is a distinct stress concentration area in the frontal part of skull. When t = 6.0 ms, there is a peak value of von Mises stress at the frontal impact, and the maximum equivalent stress is 448 MPa. Because this area is directly in contact with the ground, there is a relatively serious stress concentration.

Based on the von Mises criterion of Mcelhaney et al. [22], the fracture will occur when the von Mises stress exceeds 75 MPa. However, in this instance, the von Mises stress of the frontal bone is 103.42 MPa; therefore, there will be a relatively serious fracture of the frontal bone, which corresponds to the comminuted fracture of the frontal bone recorded in the accident investigation report. Concomitantly, the fracture of the frontal bone will also cause a dural hematoma. In addition, another part of the stress also propagates to the brain through the coupling of skull and brain tissue. When the stress wave propagates to the coupling, it will appear as attenuation. Therefore, the von Mises stress of brain tissue under the collision site is significantly lower than the von Mises stress of the frontal skull.

Fig 15 shows the stress wave propagation of the brain tissue. It can be seen that from t = 4.0 to t = 6.0 ms, the stress gradually increases in the contact area between the frontal lobe and the skull, and the area of stress change gradually increases and radiates all around. At the same time, the stress wave continues to propagate gradually to all of the brain and is reduced in the direction of collision. There is a large range of stress concentration phenomena in the frontal lobe under the collision point, in which the stress at the frontal pole has a moderate increase. When the stress wave propagates to the corpus callosum, the stress will be concentrated in a certain range. In addition, the relative movement of the skull and brain will cause the contact, friction and collision of the irregular structure of the skull base, resulting in a large stress in the local area of the skull base, and the stress at the skull base of the posterior cranial fossa is significantly greater than at other parts nearby. When t = 6.0 ms, the maximum von Mises stress of brain tissue appears on the collision side with a peak value of 99.76 kPa. Based on the brain injury criterion of Baumgartner et al. [23], when the von Mises stress of brain tissue exceeds 38 kPa, it will cause serious injury to the brain of the pedestrian, which is manifested in the death of the pedestrian in the clinic. It can be seen that the simulation results are largely consistent with the pedestrian injury in the real accident.

**Fig 16:**
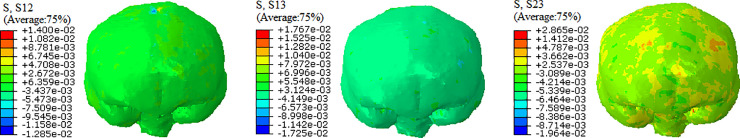
Shear stress of frontal brain tissue. **(A)** XY plane. **(B)** XZ plane. **(C)** YZ plane.

As shown in Fig 16, we can also observe the shear stress in three directions, which produces the maximum shear stress in YZ plane with the maximum value of 28.65 kPa and leads to large shear deformation of brain tissue in YZ plane. According to the tolerance limit of shear stress of Anderson et al. [24], when the shear stress of brain tissue exceeds 16 kPa, it will lead to moderate diffuse axonal injury (DAI) of the brain tissue, which is shown in the pedestrian brainstem hemorrhage recorded in the clinical pathological report. It can be seen that the simulation results are largely consistent with the pedestrian injury in the real accident.

**Fig 17:**
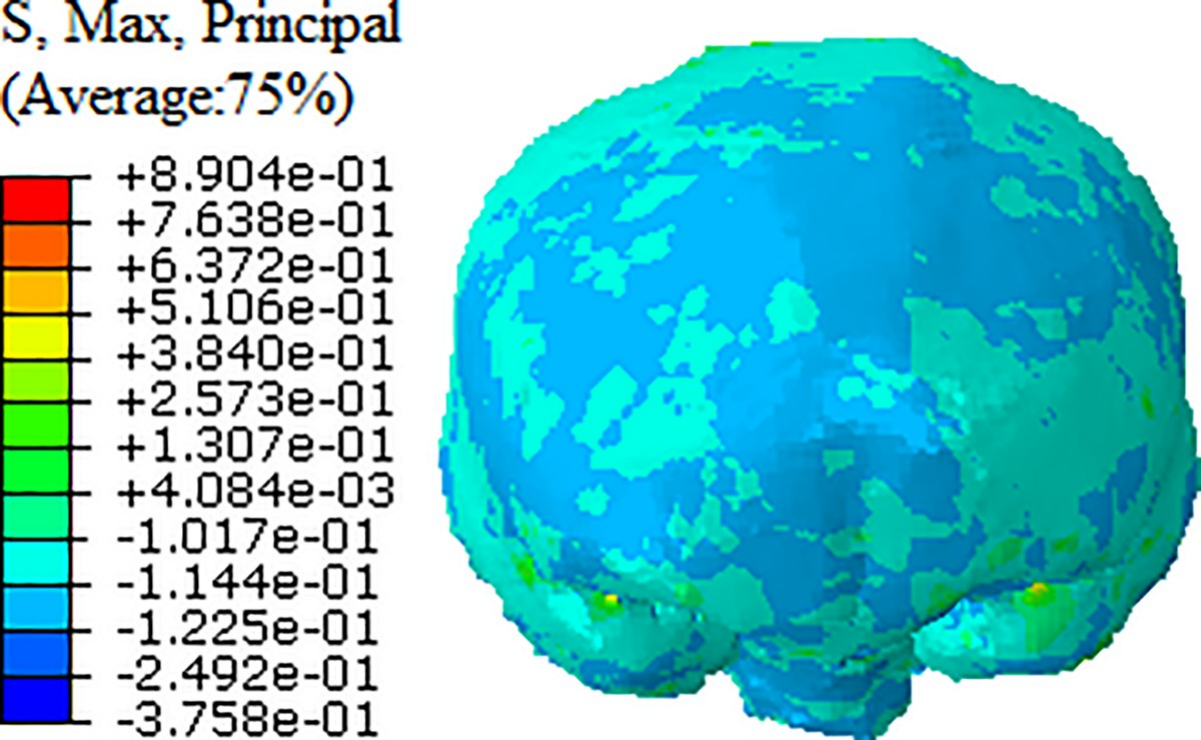
Strain of frontal brain tissue.

In addition, we can see from Fig 17 that the maximum strain value appears in the middle cranial fossa area, and the peak strain is approximately 0.890. According to the tolerance limit of Bain et al. [25], when the strain of brain tissue exceeds 0.28, there will be traumatic brain injury in the pedestrian, which is shown in the clinical pathological report as pedestrian brainstem injury.

## 4 Discussion

From the process of stress wave propagation in frontal collision, it can be found that the stress wave starts from the collision site and propagates along the skull and brain tissue in all directions. In the process of stress wave transmission, the skull and brain tissue will be damaged in different degrees. There are two primary ways to propagate stress waves. The first is from the collision side to the opposite side of the collision in the skull, that is, from the collision part of the skull, the stress gradually travels along the skull cap and skull base, and the stress concentrates on the skull base, the collision part and the skull nearby. The second way is from the collision side to the opposite side of the collision in the brain, that is, the stress wave first passes through the skull and brain, and the stress is concentrated in the brain tissue and the base of the brain.

The wave attenuation occurs when the stress wave propagates to the craniocerebral coupling; therefore, the von Mises stress of the skull at the impact site is significantly higher than that of the brain tissue at the impact site, which is consistent with the research results of He LM et al. [26]. The stress wave propagates from the brain tissue of the collision site to the brain and gradually attenuates in the process of brain tissue transmission. The stress concentration will not only appear in the skull and brain tissue of the collision side but will also exist in the opposite side from the collision. Combined with the specific injury situation of the pedestrian head, the stress concentration may be an injury mechanism of the head landing injury.

In frontal impact, the stress wave mainly propagates to the maxillofacial region, while in the multicavity structure of the maxillofacial bone, it is easy to form a stress concentration area due to the irregular reflection and transmission of the stress wave, and it also absorbs part of the impact energy. In addition, the parietal bone is arched, and the collagen fibers of its outer plate are staggered and have multidirectional distribution on different layers. Taking these fibers as scaffolds to form the bone plate, the shape of the skull top is similar to "thin shell structure", which can well disperse the external forces. Therefore, no obvious stress concentration area was found in the temporal and frontal collision of the parietal bone.

## 5 Conclusion

In this paper, a real-life collision accident is simulated. The speed and angle of pedestrian head landing moment obtained from the accident reconstruction are taken as the initial conditions, and the mechanism of pedestrian head landing injury is studied by using the brain finite element model with detailed craniofacial structure.

1. The craniocerebral injury after pedestrian head impact on the ground is related to von Mises stress and strain. The stress distribution and stress wave propagation reflect the shock response process of craniocerebral injury. It is more intuitive to see the process of craniocerebral injury. There are two main paths of stress wave propagation.
2. By reconstructing the process of brain deceleration injury caused by pedestrian impact on the ground in a case, it can be seen that the simulation results are consistent with the actual situation of brain deceleration injury. Comparing the results of finite element simulation with the injuries of pedestrians in the accident, it is found that when pedestrians collide with the ground, there is a stress concentration in the brain tissue and skull under the collision site, and there is a stress concentration area in the opposite part of the collision and the irregular part of the skull base, among which the stress in the skull site is the largest.
3. The accurate reconstruction of the collision model of pedestrian brain injury can predict the severity of injury in various parts of the human body under different working conditions and may provide a reference for further research investigating the protection of pedestrian brains and the improvement of vehicle front shapes.
